# Psychological distress in biliary tract malignancy patients: influencing factors and development of a predictive nomogram model

**DOI:** 10.3389/fpsyt.2024.1450860

**Published:** 2024-12-04

**Authors:** Zhennan Gou, Yuhua Liu, Wenjie Tang, Changming Zhou, Zhenqi Lu, Lu Wang, Wei Feng, Weiqi Xu, Jun Wang

**Affiliations:** ^1^ Department of Nursing, Shanghai Cancer Center, Fudan University, Shanghai, China; ^2^ Department of Cancer Prevention, Shanghai Cancer Center, Fudan University, Shanghai, China; ^3^ Department of Psychological Medicine, Shanghai Cancer Center, Fudan University, Shanghai, China; ^4^ Department of Hepatic Surgery, Shanghai Cancer Center, Fudan University, Shanghai, China

**Keywords:** biliary tract cancer, psycho-oncology, psychological distress, nomogram, prediction model, model validation

## Abstract

**Objective:**

This study aims to investigate the psychological distress and its influencing factors in patients with biliary tract malignant tumors, alongside the development of a predictive model.

**Methods:**

A total of 219 patients diagnosed with biliary tract malignant tumors who were admitted to the Department of Liver Surgery at Fudan University Shanghai Cancer Center from July 2021 to May 2023, were selected using a convenience sampling method. Research tools involve psychological distress management screening tools, a demographic questionnaire, self-rating anxiety and depression scales, and the Chinese version of the Memorial Symptom Assessment Scale. Bootstrap method was utilized for repeated sampling to identify relevant factors influencing psychological distress in biliary tract cancer patients. The R software was employed to create a nomogram model, and the model’s accuracy and predictive performance were assessed using the receiver operating characteristic curve (ROC) and the Hosmer-Lemeshow test.

**Results:**

The average score of psychological distress among the 219 patients was (3.91 ± 2.44), with a psychological distress detection rate of 54.8%. Regression model results indicated that factors such as the presence of distant metastasis, comorbidity with other major diseases, poor sleep quality, anxiety, and severity of anxiety and depression were the primary influencers of psychological distress.

**Conclusion:**

The detection rate of psychological distress in patients with biliary tract malignant tumors is notably high. The predictive model constructed in this study exhibits good predictive efficacy and clinical value, providing valuable reference for healthcare professionals in developing targeted intervention strategies.

## 1 Introduction

Biliary tract cancer (BTC), characterized by its high malignancy, originates from the biliary system and encompasses cholangiocarcinoma (CCA) and gallbladder carcinoma (GBC). In China, the annual incidence of new cases is approximately 113,000 (1), with a rising trend globally and notably in Shanghai (2, 3). The incidence rate is higher among women and in rural areas (4). Biliary tract malignant tumors typically manifest insidiously, often without evident symptoms in the early stages. Consequently, most patients are diagnosed in the middle or late stages, with an overall prognosis of less than 5% 5-year survival rate (5, 6) and less than 40% undergoing radical surgery (7, 8). Despite surgical intervention, the 5-year recurrence rate remains as high as 50% (9). Due to challenges in early diagnosis, poor prognosis, high recurrence rates, and treatment-related trauma and side effects, patients with BTC experience significant psychological distress alongside physical suffering.

Psychological distress, also known as psychological disturbance, encompasses cognitive, behavioral, emotional, social, spiritual, and/or physical unpleasant experiences that may hinder individuals’ ability to cope effectively with cancer, physical symptoms, and treatments. It is prevalent among cancer patients, with 20% to 52% exhibiting significant psychological distress in the United States (10), and a detection rate of 24.2% among cancer patients in China (11). Psychological factors play a vital role in the onset, progression, and outcomes of malignant tumors (12), potentially impacting prognosis and mortality rates. However, research focusing on the psychological distress of BTC patients remains scarce.

This study aims to investigate the psychological distress status in patients with biliary tract malignancies, analyze its influencing factors, and establish a nomogram prediction model. The study seeks to provide a reference and basis for the development of targeted interventions.

## 2 Participants and methods

### 2.1 Participants

From July 2021 to May 2023, a total of 219 patients diagnosed with BTC were selected through convenience sampling. Inclusion criteria: (a) Pathologically confirmed biliary tract malignant tumors; (b) Absence of verbal communication and cognitive dysfunction; (c) Age over 18 years, voluntary participation, and signed informed consent. Exclusion criteria: (a) Combined with malignant tumors of other systems; (b) Concurrent psychiatric disorders; (c) Patients concealing medical conditions. Kendall’s sample size estimation method was employed and 16 predictors were included, resulting in a minimum sample size of 192 participants considering potential sample loss and invalid questionnaires. This study was approved by the Ethics Committee of the Shanghai Cancer Center, Fudan University, with ethical approval number 2107239-16.

### 2.2 Methods

#### 2.2.1 General information questionnaire

A self-designed questionnaire was utilized to gather demographic and sociological information about the patients, including age, gender, education level, marital status, residency status, occupation, per capita monthly household income, and primary payment methods for medical care.

#### 2.2.2 Distress management screening measure

The DMSM consisted of two components: the Distress Thermometer (DT) and the Problem List (PL). The DT is a visual analog scale with 11 points ranging from 0 to 10, and a DT score exceeding 4 points signifies clinically significant psychological distress. The PL further explores specific psychological distress issues experienced by cancer patients over the past week, encompassing 39 items across four dimensions: Practical Problems (6 items), Family Problems (4 items), Emotional Problems (6 items), Physical Problems (22 items), and Spiritual/Religious Concerns (1 item). Each item is answered with a “yes” or “no” response indicating the presence or absence of the specific problem. The Cronbach’s alpha coefficients for the scale ranged from 0.711 to 0.808 (10).

#### 2.2.3 Self-rating anxiety scale and self-rating depression scale

Both SAS and SDS scales assess patients’ psychological status. The SAS comprises 20 anxiety-related items scored on a 4-point scale. A standardized score of ≥50 indicates the presence of anxiety, with severity categorized as mild (50–59), moderate (60–69), or severe (≥70). The SDS also comprises 20 depressive symptom-related items scored similarly to the SAS. A standardized score of ≥53 indicates depression, with severity classified as mild (53–62), moderate (63–72), or severe (≥73). The Cronbach’s alpha coefficients were 0.929 for the SAS and 0.963 for the SDS (13).

#### 2.2.4 Chinese version of memorial symptom assessment scale

This scale comprises 32 common symptoms experienced by cancer patients over the past week. Cronbach’s alpha coefficients for the scale range from 0.79 to (14) 0.87.

### 2.3 Statistical analysis

Data were entered and analyzed using SPSS 22.0 software. Descriptive statistics were expressed as mean ± standard deviation for continuous variables and frequency and percentage for categorical variables. Between-group comparisons were conducted using one-way ANOVA, and binary regression model parameter estimation was performed with 1,000 repeated samplings using the Bootstrap method. The regression model was visualized with a column-line graph using R 4.1.3 software, with its accuracy and validity assessed using receiver operating characteristic curve (ROC) analysis and the Hosmer-Lemeshow goodness-of-fit test. Statistical significance was set at P < 0.05.

## 3 Results

### 3.1 Patients characteristics

A total of 257 questionnaires were distributed, with 219 valid responses obtained, yielding an effective recovery rate of 85.21%. In all, 219 patients were investigated, with ages ranging from 20 to 80 years (mean ± SD: 54.62 ± 11.40 years). Patients Characteristics are showed in **Supplementary Materials**.

### 3.2 Status of psychological distress in BTC patients

The psychological distress score of the 219 patients in this study was (3.91 ± 2.44) points, with 120 patients scoring ≥4 points, resulting in a psychological distress detection rate of 54.8%. The problem list survey revealed that emotional problems were reported by 150 patients, accounting for 68.5% of those with biliary malignant tumors, which could contribute to psychological distress in patients. The causes of psychological distress reported by patients are presented in **Table 1**, with the top 10 frequencies of occurrence being sadness, worry, financial concerns, family health issues, depression, attention problems, fatigue, pain, family responsibilities, and nervousness. These results are summarized in **Table 1**.

**Table 1:** Occurrence of causes of psychological distress in BTC patients (n=219).

Items	Cases	Percent(%)
Practical Problems	111	50.7%
Transportation	8	3.7%
Insurance/Financial	36	16.4%
Work	2	0.9%
Housing	18	8.2%
Child care	17	7.8%
Treatment decisions	30	13.7%
Family Problems	68	31.1%
Dealing with children	15	6.8%
Dealing with partner	12	5.5%
Ability to have children	11	5.0%
Family health issues	30	13.7%
Emotional Problems	150	68.5%
Depression	28	12.8%
Fears	16	7.3%
Nervousness	18	8.2%
Sadness	45	20.5%
Worry	39	17.8%
Loss of interest in usual activities	4	1.8%
Physical Problems	176	80.4%
Appearance	6	2.7%
Bathing/dressing	1	0.5%
Changes in urination	2	0.9%
Constipation	10	4.6%
Diarrhea	4	1.8%
Eating	5	2.3%
Fatigue	25	11.4%
Feeling swollen	5	2.3%
Fevers	6	2.7%
Getting around	8	3.7%
Indigestion	17	7.8%
Memory/concentration	28	12.8%
Mouth sores	4	1.8%
Nausea	5	2.3%
Nose dry/congested	0	0.0%
Pain	18	8.2%
Sexual	0	0.0%
Skin dry/itchy	12	5.5%
Sleep	8	3.7%
Substance use	1	0.5%
Tingling in hands/feet	0	0.0%
Memory/concentration	11	5.0%
Spiritual/religious Concerns	9	4.1%

### 3.3 A univariate analysis of factors influencing psychological distress in BTC patients

The results of the analysis indicate the potential factors influencing the psychological distress among BTC patients. These factors include age, presence of underlying diseases, presence of distant metastases, severity of anxiety and depression measured by SAS and SDS, pain, poor sleep quality, feelings of sadness, anxiety, and irritability (P < 0.05). Refer to **Table 2** for details.

**Table 2:** Univariate analysis of factors influencing psychological distress in BTC patients (n=219).

Item	Cases	The Score of DT	F-value	P-value
Age				
<60	149	4.26 ± 2.60	9.90	0.02
≥60	70	3.17 ± 1.88		
The presence of comorbidities with other major underlying illnesses				
Y	117	4.26 ± 2.37	5.33	0.02
N	102	3.51 ± 2.47		
The presence of distant metastases				
Y	47	6.81 ± 1.83	136.8	<0.001
N	172	3.12 ± 1.94		
Degree of anxiety				
No	141	3.06 ± 2.14	24.13	<0.001
Low	55	5.05 ± 1.97		
Moderate	19	6.21 ± 2.23		
High	4	7.50 ± 1.92		
Degree of depression				
No	100	2.48 ± 1.98	59.87	<0.001
Low	70	4.17 ± 1.61		
Moderate	37	5.84 ± 1.82		
High	12	8.42 ± 1.38		
Pain				
Y	125	4.58 ± 2.37	23.74	<0.001
N	94	3.03 ± 2.26		
Difficulty sleeping				
Y	109	5.10 ± 2.34	67.01	<0.001
N	110	2.74 ± 1.91		
Feeling sad				
Y	80	5.65 ± 2.11	89.95	<0.001
N	139	2.91 ± 2.02		
Feeling nervous				
Y	61	5.13 ± 2.53	23.22	<0.001
N	158	3.44 ± 2.24		
Feeling irritable				
Y	53	4.72 ± 2.65	7.83	0.006
N	166	3.66 ± 2.32		

Only statistically significant variables are listed.

### 3.4 Regression analysis of factors affecting psychological distress in patients with BTC

Results of the regression analysis based on the Bootstrap method with 1000 repeated samplings indicate that distant metastasis, other major underlying diseases, poor sleep, anxiety, and depression levels were independent risk factors for psychological distress in BTC patients (see **Table 3**). In this analysis, we took the presence or absence of psychological distress in patients as the dependent variable, and the items with differences in the univariate analysis results as the independent variables. The assignment method of each independent variable is shown in **Supplementary Materials**.

**Table 3:** Binary regression model obtained by Bootstrap method (n=219).

Items	β	B	SE	P	OR(95%CI)
(Constant)	-3.002	-0.189	0.533	0.001	
The presence of distant metastases	20.525	0.074	0.472	0.001	8.20(3.63,22.81)*10^8
The presence of comorbidities with other major underlying illnesses	1.034	0.092	0.472	0.016	2.81(1.26,8.50)
Difficulty sleeping	1.11	0.082	0.49	0.01	3.03(1.31,8.94)
Feeling nervous	1.154	0.084	0.503	0.011	3.17(1.25,9.20)
Degree of anxiety	1.274	0.069	0.509	0.001	3.58(1.43,10.77)
Degree of depression	1.493	0.089	0.364	0.001	4.45(2.50,10.14)

### 3.5 Construction and validation of a predictive model for psychological distress risk in BTC patients

**Figure 1:**
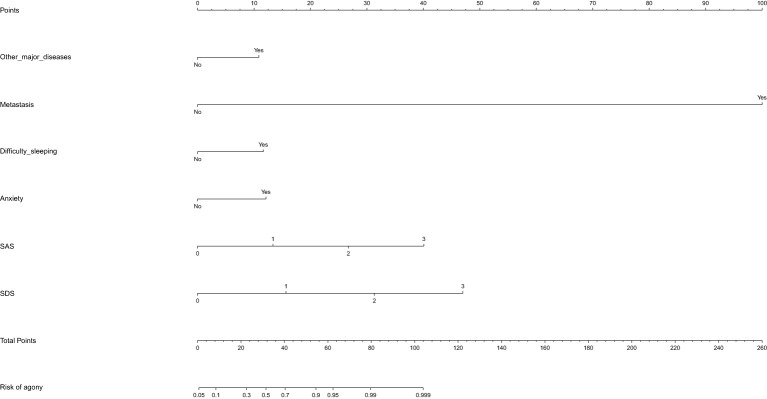
A nomogram prediction model of factors influencing psychological distress in BTC patients.

**Figure 2:**
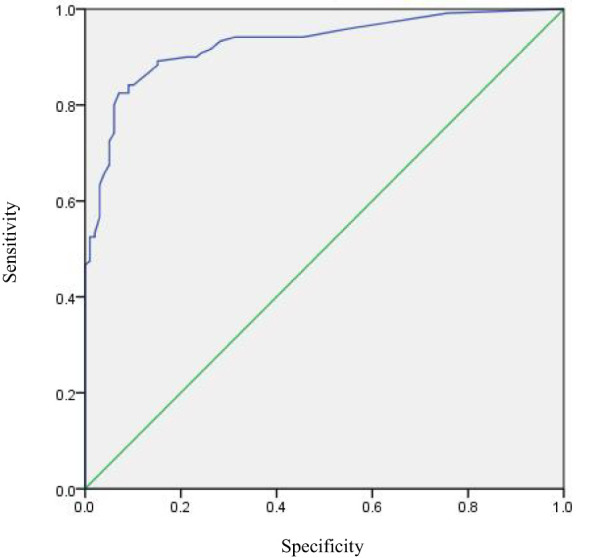
ROC curve of the predictive model of factors influencing psychological distress in BTC patients.

**Figure 3:**
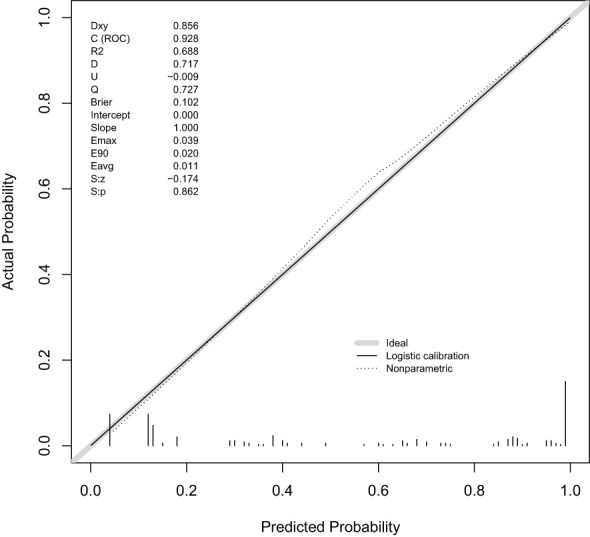
Calibration curves of the predictive model of factors influencing psychological distress in BTC patients.

A predictive model was constructed based on the regression analysis results: Logit (P) = -3.002 + 20.525 × the presence of distant metastases + 1.034 × the presence of comorbidities with other major underlying illnesses + 1.11 × difficulty sleeping + 1.154 × feeling nervous + 1.274 × degree of anxiety + 1.493 × degree of depression. The nomogram prediction model was visualized using R software, as depicted in **Figure 1**. The area under the ROC curve (AUC) of the prediction model was 0.928 (95% CI: 0.894~0.962), indicating its strong predictive value (see **Figure 2**). The Hosmer-Lemeshow goodness-of-fit test yielded a chi-square value of 0.688 with a P-value of 0.862 (>0.05), suggesting good model fit. The calibration curve slope approaching 1 further confirmed the model’s accuracy (see **Figure 3**).

## 4 Discussion

The results of this study reveal that the Distress Thermometer (DT) score among 219 BTC patients was (3.91 ± 2.44) points, with a clinically significant psychological distress detection rate of 54.8%. This rate is notably higher than the norm of 24.2% reported among Chinese cancer patients (11) and even higher than that in hepatocellular carcinoma patients as reported by Li-Ru Pan et al. (36.67%) (15). The psychological distress among BTC patients in this study is found to be considerably elevated. Patients with malignant tumors often experience varying degrees of negative emotions, such as anxiety and depression. BTC, characterized by its insidious onset, high malignancy, high recurrence and metastasis rates, and low survival rate, brings about significant psychological distress. Apart from the psychological trauma stemming from the disease diagnosis, patients also grapple with the fear of disease progression and death, leading to considerable psychological pain. Moreover, symptoms like pain, abdominal distension, and loss of appetite further exacerbate patients’ psychological distress, particularly in the context of the prevalent Chinese cultural notion that attaches more importance to life rather than death. Hence, anticipatory sadness about death and weaker psychological resilience among patients could exacerbate psychological distress. Consequently, sadness (20.5%) and worry (17.8%) emerged as the most frequently reported problems among BTC patients in the Problem List (PL) survey. Additionally, insurance/financial problems (16.4%) were also prevalent among BTC patients. Despite the ongoing development and improvement of China’s health insurance system, the cost of cancer treatment remains a significant burden for patients, further compounding their psychological distress.

The overall detection rate of clinically significant psychological distress among BTC patients in this study is much higher than that among patients with other types of cancer. Healthcare professionals need to pay greater attention to the psychological well-being of this population, identify psychological distress at an early stage, and implement appropriate interventions to enhance their quality of life. However, the detection rate of clinically significant psychological distress among BTC patients in this study is not comparable to that reported among cancer patients in North America and Vietnam (16, 17), indicating potential differences in screening tools, ethnicity, economic status, culture, national conditions, and cognitive patterns. Therefore, future screening and intervention efforts should consider factors beyond the disease itself.

Furthermore, the study finds that the presence of distant metastases may be a decisive factor in causing psychological distress among BTC patients. The 5-year survival rate for BTC patients is only about 5% (5, 6), and metastasis signifies increased malignancy, severe symptoms, challenging treatment, and a shorter survival period, leading to both physical and psychological distress. Additionally, comorbidities with other major underlying diseases, difficulty sleeping, feeling nervous, and the degree of anxiety or depression were identified as contributors to psychological distress among BTC patients. Notably, anxiety and depression exerted the most significant impact on psychological distress. The presence of anxiety or comorbidity of anxiety and depression often seem to lead to higher psychological distress among patients, as stated in the NCCN guidelines (10). Moreover, the diagnosis of BTC exacerbated existing health issues, compounding the psychological burden on patients. Persistent sleep disorders, common among cancer patients, can worsen fatigue, weaken immunity, and trigger negative emotions (18, 19), further exacerbating psychological distress and affecting prognosis and quality of life.

### 4.1 Clinical implications

To address these findings, once a patient is diagnosed with BTC, healthcare professionals should assess whether the patient has relevant risk factors. Medical professionals are encouraged to intervene actively in BTC patients with distant metastases, strengthen disease education and psychological support to alleviate psychological distress, help them better cope with the challenges and uncertainties in the treatment process, and improve their quality of life. Additionally, personalized interventions targeting the identified risk factors should be provided to patients without distant metastases to mitigate psychological distress. Relevant professional measurement tools can be used at various stages of the patient’s illness to accurately identify whether there are symptoms that may lead to psychological distress (such as the SAS/SDS used in this study), and the predictive model constructed in this study can be used to predict the likelihood of psychological distress in patients and provide targeted intervention measures, such as using relaxation therapy or appropriate medication for patients with sleeping disorders. In addition to interventions targeting a single variable, we also recommend implementing comprehensive intervention strategies to comprehensively improve the psychological condition of patients. This includes various intervention measures such as psychological counseling, social support, pain management, and necessary medication treatment. Therefore, the participation of multidisciplinary medical team including oncologists, psychotherapists, pharmacists, and oncology specialist nurses is also important in order to better address patients’ different symptoms or problems. Future research may attempt to further validate the clinical significance and intervention effects of the predictive factors we have discovered. Especially, by designing prospective studies with larger sample sizes and longer time spans, we can gain a deeper understanding of the dynamic changes in psychological distress and its interactions with clinical factors. Moreover, more potential intervention measures can be explored and their long-term impact on the psychological status of patients can be evaluated.

The study constructed a predictive model using regression analysis and Bootstrap sampling to identify six factors influencing psychological distress among BTC patients. After the development of the model, we conducted rigorous internal validation to ensure its stability and reliability. Internal validation was conducted using bootstrap resampling method, estimating the predictive performance of the model through 1,000 repeated samples. The resulting model exhibited a strong ability to accurately identify factors contributing to psychological distress, as evidenced by the area under the ROC curve of 0.928. The model’s calibration was also deemed satisfactory, as indicated by the Hosmer-Lemeshow test results. This predictive model enables healthcare professionals to identify BTC patients at risk of psychological distress early on and deliver tailored interventions accordingly. In addition, in order to further enhance the generalization ability of the model, we plan to conduct external validation in the future. External validation will use datasets from different medical institutions or regions to evaluate the predictive performance of the model in different populations. Although external validation has not yet been completed, we believe that through rigorous internal validation and a reasonable model construction process, our predictive column chart will maintain a certain level of accuracy and stability in different scenarios. In the future, we will actively seek opportunities for cooperation to obtain more external data for verification and further improve and optimize our model.

### 4.2 Study limitations

However, it’s important to note the limitations of this study. Firstly, this study was conducted in a single ward of a tertiary specialized hospital with a small sample size. We acknowledge that the convenience sampling method used in this study may have the risk of selection bias, which may result in insufficient representativeness of the sample. Although we strive to ensure sample diversity, it cannot be denied that this method may not fully cover all types of biliary malignant tumor patients, such as young patients. To mitigate this potential impact, we considered sample features during the data analysis phase and attempted to reduce bias through weighted adjustments. However, we also realize that these efforts may not completely eliminate the influence of selection bias. Future research should prioritize the use of more representative sampling methods, such as random sampling or stratified sampling, to further improve the reliability and generalizability of the study. Furthermore, due to the lack of external validation, the generalizability of our research results may be limited. Future research may benefit from multicenter, large-scale studies, and consider patients from other countries and ethnicities. Secondly, our study only used a cross-sectional survey method to investigate the psychological distress of BTC patients which may unable to demonstrate the relationship between psychological distress and time. Employing longitudinal studies could offer insights into the dynamic trajectory of patients’ psychological distress over time. Moreover, due to certain limitations, this study failed to collect some relating data such as detailed pathological information, the duration of BTC diagnosis or treatment history, symptom duration and history. Including these variables may provide additional insights into distress predictors. Therefore, the effectiveness of this prediction model may be affected to some extent. Thirdly, In terms of statistical methods, although Bootstrap sampling has significant advantages in improving analysis robustness, there are also some potential limitations. Overfitting is an important issue. In the process of model construction, excessive reliance on the resampling results of Bootstrap sampling may result in the model performing well on the training set but poorly on new, unseen data. In addition, Bootstrap sampling may also be affected by sample representativeness. If the original sample itself is not representative, the resampled data obtained through Bootstrap sampling may not fully reflect the overall characteristics, thereby affecting the accuracy of the results. Therefore, in future research, we should strictly follow scientific methods and standards to ensure the representativeness of the samples and the reliability of the data. In addition, the Bootstrap sampling method may face computational efficiency issues when dealing with large-scale datasets. Although the data volume is moderate in this study, the computational cost of Bootstrap sampling may significantly increase as the data size increases. Therefore, in future research, we should explore more efficient sampling and modeling methods to address the challenges of the big data era.

## 5 Conclusion

The findings of this study indicate a high level of clinically significant psychological distress detection rate among BTC patients, with the presence of distant metastases, comorbidities with other major underlying diseases, anxiety, poor sleep, and the severity of anxiety or depression being the primary influencing factors. Healthcare professionals can utilize the predictive model developed in this study to identify high-risk individuals prone to psychological distress and implement timely, targeted interventions aimed at alleviating the causes of psychological distress, thereby reducing psychological distress and enhancing patients’ quality of life.

## Data Availability

The raw data supporting the conclusions of this article will be made available by the authors, without undue reservation.

## Supplementary material

Supplementary Table 1 Characteristics of BTC patients (n=219).

Supplementary Table 2 The assignment method of each independent variable in the binary regression model.
