# Supplementary material for: Psychological distress in biliary tract malignancy patients: influencing factors and development of a predictive nomogram model
Source: Front Psychiatry. 2024 Dec 4;15:1450860. doi: 10.3389/fpsyt.2024.1450860 (PMC11652499; doi:10.3389/fpsyt.2024.1450860)
Supplement: Supplementary Table 1 — Characteristics of BTC patients (n=219). [file Table1.docx]

1. Characteristics of BTC Patients

Table 1: Characteristics of BTC patients（n=219）

| **Items** | **Cases** | **Percent（%）** |
| --- | --- | --- |
| Sex |  |  |
| Male | 135 | 61.6% |
| Female | 84 | 38.4% |
| Education Level |  |  |
| Junior high school or below | 121 | 55.3% |
| Senior high school or vocational education | 71 | 32.4% |
| Bachelor's degree or higher | 27 | 12.3% |
| Marital status |  |  |
| Unmarried | 5 | 2.3% |
| Married | 201 | 94.1% |
| Divorced | 6 | 2.7% |
| Widowed | 7 | 3.2% |
| Occupation |  |  |
| Employed in enterprises or institutions | 38 | 17.4% |
| Self-employed | 26 | 11.9% |
| Retired | 64 | 29.2% |
| Farmers | 52 | 23.7% |
| Other occupations | 39 | 17.9% |
| Per capita monthly household income |  |  |
| <2,000 yuan | 41 | 18.7% |
| 2,000-5,000 yuan | 92 | 42% |
| 5,001-8,000 yuan | 40 | 18.3% |
| 8,001-10,000 yuan | 18 | 8.2% |
| ≥10,001 yuan | 28 | 12.8% |
| Living arrangements |  |  |
| Alone | 10 | 4.6% |
| With a spouse | 87 | 39.7% |
| In two-generation households | 86 | 39.3% |
| In three-generation households | 33 | 15.1% |
| Others | 3 | 1.4% |
| Main payment methods for medical expenses |  |  |
| Medical insurance | 143 | 65.3% |
| Out of pocket | 31 | 14.2% |
| Commercial insurance | 2 | 0.9% |
| Agricultural insurance | 43 | 19.6% |
| Religious beliefs |  |  |
| Yes | 17 | 7.8% |
| No | 202 | 92.2% |
