# Supplementary material for: Psychological distress in biliary tract malignancy patients: influencing factors and development of a predictive nomogram model
Source: Front Psychiatry. 2024 Dec 4;15:1450860. doi: 10.3389/fpsyt.2024.1450860 (PMC11652499; doi:10.3389/fpsyt.2024.1450860)
Supplement: Supplementary Table 2 — The assignment method of each independent variable in the binary regression model. [file Table2.docx]

1. The assignment method of each independent variable in the binary regression model

Table 2: The assignment method of independent variables

| **Items** | **Assignment Methods** |
| --- | --- |
| Age | <60=1；≥60=2 |
| The presence of comorbidities with other major underlying illnesses | Y=1；N=0 |
| The presence of distant metastases | Y=1；N=0 |
| Degree of anxiety | No=0；Low=1；Moderate=2；High=3 |
| Degree of depression | No=0；Low=1；Moderate=2；High=3 |
| Pain | Y=1；N=0 |
| Difficulty sleeping | Y=1；N=0 |
| Feeling sad | Y=1；N=0 |
| Feeling nervous | Y=1；N=0 |
| Feeling irritable | Y=1；N=0 |
